# Sequential Organ Failure Assessment Score Can Predict Mortality in Patients with Paraquat Intoxication

**DOI:** 10.1371/journal.pone.0051743

**Published:** 2012-12-14

**Authors:** Cheng-Hao Weng, Ching-Chih Hu, Ja-Liang Lin, Dan-Tzu Lin-Tan, Wen-Hung Huang, Ching-Wei Hsu, Tzung-Hai Yen

**Affiliations:** 1 Department of Nephrology, Chang Gung Memorial Hospital, Linkou Medical Center, Taipei, Taiwan; 2 Department of Hepatogastroenterology and Liver Research Unit, Chang Gung Memorial Hospital, Keelung, Taiwan; 3 Chang Gung University, College of Medicine, Taoyuan, Taiwan; University of Sao Paulo Medical School, Brazil

## Abstract

**Introduction:**

Paraquat poisoning is characterized by multi-organ failure and pulmonary fibrosis with respiratory failure, resulting in high mortality and morbidity. The objective of this study was to identify predictors of mortality in cases of paraquat poisoning. Furthermore, we sought to determine the association between these parameters.

**Methods:**

A total of 187 patients were referred for management of intentional paraquat ingestion between January 2000 and December 2010. Demographic, clinical, and laboratory data were recorded. Sequential organ failure assessment (SOFA) and acute kidney injury network (AKIN) scores were collected, and predictors of mortality were analyzed.

**Results:**

Overall hospital mortality for the entire population was 54% (101/187). Using a multivariate logistic regression model, it was found that age, time to hospitalization, blood paraquat level, estimated glomerular filtration rate at admission (eGFR_ first day_), and the SOFA_48-h_ score, but not the AKIN_48-h_ score, were significant predictors of mortality. For predicting the in-hospital mortality, SOFA_48-h_ scores displayed a good area under the receiver operating characteristic curve (AUROC) (0.795±0.033, *P*<0.001). The cumulative survival rate differed significantly between patients with SOFA_48-h_ scores <3 and those ≥3 (*P*<0.001). A modified SOFA (mSOFA) score was further developed by using the blood paraquat level, and this new score also demonstrated a better AUROC (0.848±0.029, P<0.001) than the original SOFA score. Finally, the cumulative survival rate also differed significantly between patients with mSOFA scores <4 and ≥4 (*P*<0.001).

**Conclusion:**

The analytical data demonstrate that SOFA and mSOFA scores, which are based on the extent of organ function or rate of organ failure, help to predict mortality after intentional paraquat poisoning.

## Introduction

Due to ease of access, pesticides [1] and herbicides [2] are commonly ingested in Taiwan, both intentionally and by accident. Paraquat is a popular bipyridal herbicide with a good safety record when used properly. However, the lethal toxicity of this compound leads to a high mortality rate (60–80%). After ingestion of approximately 40 mL of a 24% solution of paraquat, patients normally die within several hours to days from multiple organ failure. After ingesting approximately 16 mL, patients experience moderate to severe poisoning and die within 1–2 weeks from pulmonary fibrosis and severe hypoxemia [3], [4]. Many treatment modalities have been developed for paraquat poisoning, including adsorbents, hypo-oxygenation, lung radiotherapy [5], prolonged extracorporeal detoxification [6], and lung transplantation. However, the efficacies of these therapeutic methods remain uncertain.

**Table 1:** SOFA scoring system.

	0	1	2	3	4
PaO_2_/FiO_2_	>400	301–400	201–300	101–200 withrespiratory support	≤100 with respiratory support
Platelets (1000/µL)	>150	101–150	51–100	21–50	≤20
Bilirubin (mg/dL)	<1.2	1.2–1.9	2.0–5.9	6.0–11.9	>12.0
Hypotension	MAP≥70 mmHg	MAP<70 mmHg	Dopamine 5or dobutamine(any dose)*	Dopamine >5or epi ≤0.1or norepi ≤0.1*	Dopamine >15or epi >0.1or norepi >0.1*
GCS	15	13–14	10–12	6–9	<6
Cr (mg/dL) or UO	<1.2	1.2–1.9	2.0–3.4	3.5–4.9 or <500 mL/d	>5.0 or <200 mL/d

**Table 2:** AKIN scoring system.

Category	Serum Cr criteria	Urine output criteria
Stage 1	Increase in serum Cr of ≥0.3 mg/dL or increase to≥150% to 200% (1.5 to 2-fold) from baseline	<0.5 mL/kg/h for more than 6 h
Stage 2	Increase in serum Cr to >200% to 300%(>2 to 3-fold) from baseline	<0.5 mL/kg/h for more than 12 h
Stage 3	Increase in serum Cr to >300% (3-fold) frombaseline (or serum Cr of ≥4.0 mg/dL with an acute increaseof at least 0.5 mg/dL	<0.3 mL/kg/h for 24 h or anuria more for 12 h

* Adrenergic agents administered for at least 1 h (doses are given in µg/kg per minute). Abbreviations. PaO_2_: partial pressure of oxygen in arterial blood, FiO_2_: fractional inspired oxygen, MAP: mean arterial pressure, epi: epinephrine, norepi: norepinephrine, GCS: Glasgow Coma Scale score, Cr: creatinine, UO: urine output.

At our hospital, all paraquat patients are usually treated using a standard detoxification protocol [2]–[4], [7]–[10]. This protocol consists of repeated pulses of methylprednisolone and cyclophosphamide, followed by prolonged dexamethasone therapy. With this approach, it was demonstrated that both respiratory function and blood oxygen concentrations in most patients returned to near-normal levels in 3–6 months [11]. Methylprednisolone, cyclophosphamide, and dexamethasone are potent anti-inflammatory medical therapies. Therefore, it is the severe inflammation (not lung fibrosis) that plays a critical role in producing lethal hypoxemia after paraquat poisoning.

Regarding patient prognosis, Sawada *et al.* showed that a severity index of paraquat poisoning (SIPP) may be a good clinical predictor of mortality [12]. However, many hospitals do not have the necessary facilities for measuring serum paraquat levels. Huang *et al.* [13] demonstrated that an Acute Physiology And Chronic Health Evaluation II (APACHE II) score of >13, calculated 24 h after admission, could predict in-hospital mortality with 67% sensitivity and 94% specificity. Furthermore, the APACHE II system yielded better discriminative power than SIPP score, plasma paraquat concentration, or estimated paraquat ingestion dosage [13]. Nevertheless, the APACHE II score does not include parameters reflecting liver damage, which are a major complication of paraquat poisoning [10]. Furthermore, calculation of the APACHE II score is complex and not suitable for typical hospital inpatients, such as the paraquat patients assessed in our study. Both the Simplified Acute Physiology Score II (SAPS II) [14] and Expanded Simplified Acute Physiology Score II (SAPS IIe) are also commonly used in intensive care units (ICUs) for predicting mortality. Min *et al*. [15] showed that SAPS II or SAPS IIe, calculated immediately after arrival at the emergency department, may be helpful in predicting outcome after acute paraquat poisoning. However, the SAPS II or SAPS IIe systems are limited by how they are calculated, since the measurement of the ratio of partial pressure of oxygen in arterial blood to the fraction of inspired oxygen (PaO_2_/FiO_2_) is only applicable to patients undergoing mechanical ventilation. Thus, it is absolutely unsuitable for spontaneously breathing patients. On the other hand, the SOFA score has been extensively used to predict the outcome of ICU patients [16] because the measurement of PaO_2_/FiO_2_ is not limited to mechanically ventilated patients, as in the SAPS II or SAPS IIe system. The calculation is simple and involves only PaO_2_/FiO_2_, platelet count, serum bilirubin level, hypotension, Glasgow Coma Score, and serum creatinine or urine output.

Paraquat poisoning is characterized by multi-organ failure and pulmonary fibrosis leading to respiratory failure, with acute kidney injury being the major cause of mortality [10]. Despite this, the prognostic significance of the AKIN scoring system [17] has not been previously studied. Consequently, the objective of this study was to identify predictors of mortality (clinical features, physiological markers, and SOFA and AKIN scores) in cases of paraquat poisoning. Furthermore, we sought to identify potential associations between these parameters.

**Table 3:** Comparison of baseline demographics and clinical characteristics between survivors and non-survivors (n = 187).

Parameter	All (n = 187)	Survivors (n = 86)	Non-survivors (n = 101)	*P*
Age (year)	42.1±15.4	36.2±12.4	47.1±16.0	<0.001
Gender (male/female)	145/42	65/21	80/21	0.6
Time to hospitalization (days)	13.5±21.1	19.1±26.9	8.7±12.9	0.001
Estimated ingestion amount (mL)	80.9±104.7	56.3±65.7	101.9±125.5	0.002
Blood paraquat level_ first day_ (ppm)	4.8±5.6	1.4±2.0	7.6±6.1	<0.001
Cr level (mg/dL)_ first day_	1.6±1.4	1.3±1.2	1.8±1.6	0.001
AST_ first day_ level (U/L)	137.5±156.1	54.4±37.3	190.0±179.6	0.005
ALT_ first day_ level	126.8±114.1	116.8±62.8	135.5±144.9	0.436
Bilirubin _first day level_ (U/L)	2.7±2.6	1.3±0.7	3.5±3.0	0.001
PaO_2 first day_ (mmHg)	84.9±19.0	86.4±12.2	83.7±23.2	0.343
AaDO_2 48-h_ (mmHg)	46.6±28.3	29.8±20.1	61.0±26.4	<0.001
PaCO_2__48-h_ (mmHg)	33.7±12.4	38.7±12.7	29.4±10.4	<0.001
PaO_2__48-h_ (mmHg)	61.3±24.9	72.1±18.5	52.1±25.9	<0.001
HCO_3_^−^_48-h_ (meq/dL)	19.9±6.8	24.8±3.5	15.7±6.1	<0.001
PaO_2_/FiO_2__first day_	424.6±94.8	431.8±61.0	418.56±116.0	0.322
PaO_2_/FiO_2__48-h_	291.8±119.0	343.3±88.0	247.1±124.6	<0.001
PLT _first day_ (1000/µL)	241.2±67.4	240.2±66.3	242.1±68.5	0.843

Abbreviations. AST: aspartate transaminase, AST_ first day_: AST at admission, ALT_ first day_: alanine transaminase at admission, Bilirubin_ first day_: bilirubin at admission, PaO_2__first day_: partial pressure of oxygen in arterial blood at admission, PaCO_2__48-h_: partial pressure of carbon dioxide in the blood 48 h after admission, PLT _first day_: platelet count at admission, AaDO_2_: alveolar-arterial differences in oxygen tension, Cr: creatinine.

**Table 4:** Comparison of AKIN and SOFA scores between survivors and non-survivors (n = 187).

	All patients (n = 187)	Survivors (n = 86)	Non-survivors (n = 101)	*P* value
AKIN_48-h_ (stage 0/1/2/3)	99/48/16/24	60/17/4/5	39/31/12/19	<0.001
SOFA_48-h_	3±2	2±2	4±2	<0.001
mSOFA	4±2	2±2	5±2	<0.001

Abbreviations. AKIN: acute kidney injury network, SOFA: sequential organ failure assessment, mSOFA: modified sequential organ failure assessment.

## Materials and Methods

This retrospective observational study complied with the guidelines of the Declaration of Helsinki and was approved by the Medical Ethics Committee of Chang Gung Memorial Hospital, a tertiary referral center located in the northern part of Taiwan. Since this study involved retrospective review of existing data, approval from the Institutional Review Board was obtained, but without specific informed consent from patients [1]. However, informed consent regarding risks associated with acute paraquat poisoning and all treatment modalities (particularly charcoal hemoperfusion) was obtained from all patients upon their initial admission. Furthermore, not only were all data securely protected (by delinking identifying information from the main data sets) and made available only to investigators, but they were also analyzed anonymously. The Institutional Review Board of Chang Gung Memorial Hospital specifically waived the need for consent for these studies. Finally, all primary data were collected according to procedures outlined in epidemiology guidelines that strengthen the reporting of observational studies.

**Table 5:** Comparison of calibration and discrimination power of AKIN and SOFA scoring methods for predicting mortality (n = 187).

	Calibration			Discrimination		
	Hosmer – Lemeshow goodness of fit	*df*	*P*	AUROC ± SE	95% CI	*P*
AKIN_48-h_	0.0	2	1.0	0.671±0.039	0.594–0.748	<0.001
SOFA_48-h_	5.582	5	0.349	0.795±0.033	0.31–0.860	<0.001
mSOFA_48-h_	0.0	6	1.0	0.848±0.029	0.791–0.904	<0.001

Abbreviations. *df*: degree of freedom, SE: standard error, CI: confidence interval, AKIN: acute kidney injury network, SOFA: sequential organ failure assessment, mSOFA: modified sequential organ failure assessment.

### Patients

A total of 187 patients were referred for management of intentional paraquat ingestion between January 2000 and December 2010. Diagnoses of paraquat poisoning were based on clinical history, physical and laboratory examinations (especially urine sodium dithionite reaction), and confirmed via blood test (spectrophotometry; Hitachi, Tokyo, Japan) [18]. The urine sodium dithionite test was based on the reduction of paraquat by sodium thionite under alkaline conditions to form stable, blue-colored radical ions. Generation of a strong navy or dark blue color generally indicates significant paraquat ingestion and often forebodes a poor prognosis [2]–[4], [7]–[10].

### Inclusion and Exclusion Criteria

Patients were included in this study if they were older than 18 years of age and had urine paraquat tests that showed dark or navy blue coloring (>5 ppm). Patients were excluded from the study if the paraquat exposure was dermal [19] or intravascular [20]. They were also excluded if they did not have detectable paraquat levels in their urine and blood or if they had major comorbidities, such as cancer or heart, lung, renal, or liver diseases. The diagnoses of major comorbidities were based on detailed clinical, physical, and laboratory examinations. Patients with pre-existing serum creatinine levels >1.4 mg/dL or alanine aminotransferase (ALT) levels >36 mg/dL or total bilirubin levels >3 mg/dL were also excluded.

**Figure 1:**
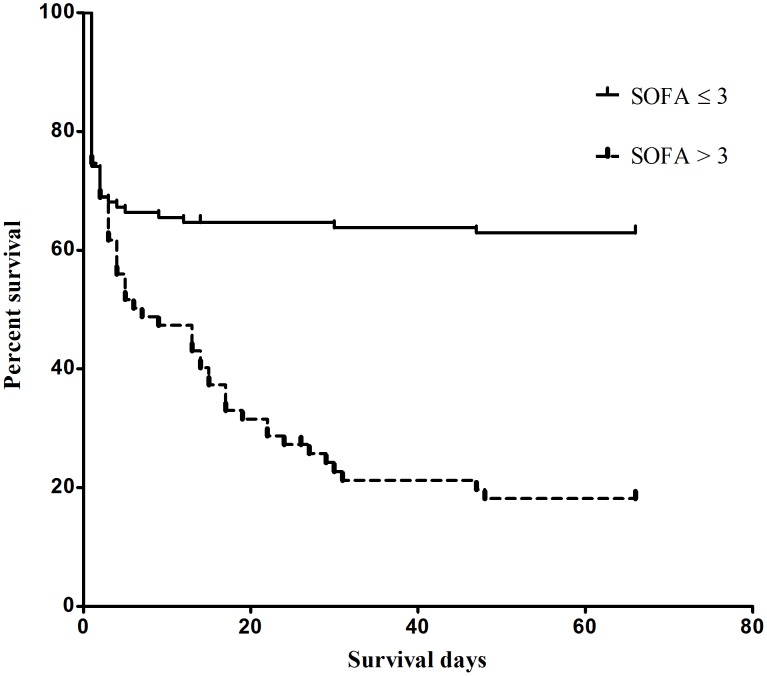
Cumulative survival rates based on SOFA _48-h_ score. Abbreviation. SOFA: sequential organ failure assessment.

**Table 6:** Analysis of mortality using univariate and multivariate logistic regression models (n = 187).

Parameter	β Coefficient	SE	Odds ratio (95% CI)	*P*
*Univariate*				
Age (year)	0.032	0.006	1.003 (1.020–1.045)	<0.001
Time to hospitalization (days)	−0.020	0.007	0.980 (0.967–0.994)	0.004
Estimated ingestion amount (mL)	0.002	0.001	1.002 (1.001–1.004)	<0.001
Blood paraquat level_ first day_ (ppm)	0.133	0.015	1.143 (1.109–1.177)	<0.001
AaDO_2__first day_ (mmHg)	0.019	0.006	1.020 (1.007–1.032)	0.002
PH _48-h_	−3.400	0.495	0.033 (0.013–0.088)	<0.001
PaO_2__48-h_ (mmHg)	−0.019	0.004	0.981 (0.972–0.989)	<0.001
PaCO_2__48-h_ (mmHg)	−0.067	0.012	0.935 (0.913–0.958)	<0.001
HCO_3__48-h_ (meq/dL)	−0.146	0.015	0.864 (0.839–0.890)	<0.001
AaDO_2__48-h_ (mmHg)	0.022	0.004	1.022 (1.015–1.030)	<0.001
eGFR _first day_ (mL/min)	−0.012	0.003	0.988 (0.983–0.994)	<0.001
AKIN _48-h_	0.246	0.084	1.279 (1.086–1.507)	0.003
SOFA _48-h_	0.193	0.036	1.213 (1.130–1.303)	<0.001
*Multivariate*				
Age (year)	0.013	0.007	1.013 (1.000–1.026)	0.049
Time to hospitalization (days)	−0.035	0.010	0.965 (0.947–0.984)	<0.001
Blood paraquat level _first day_ (ppm)	0.098	0.017	1.103 (1.066–1.140)	<0.001
eGFR _first day_ (mL/min)	−0.018	0.004	0.982 (0.974–0.991)	<0.001
SOFA _48-h_	0.101	0.042	1.106 (1.019–1.201)	0.016

Abbreviations: AKIN: acute kidney injury network, SOFA: sequential organ failure assessment, SE: standard error, CI: confidence interval, AaDO_2_: alveolar–arterial differences in oxygen tension, PaO_2__48-h_: partial pressure of oxygen in arterial blood 48 h after admission, PaCO_2__48-h_: partial pressure of carbon dioxide in the blood 48 h after admission, eGFR _first day_: estimated glomerular filtration rate at admission.

### SOFA and AKIN Scores

The following data were prospectively collected: baseline demographics; SOFA [16] and AKIN score [17] 48 h after admission (SOFA_48-h_ and AKIN_48-h_), duration of hospitalization, and outcomes. As shown in Table 1 and 2, the SOFA score [16] consists of six variables, each representing an organ system. Each organ system is assigned a point value from 0 (normal) to 4 (high degree of dysfunction/failure). The AKIN criteria [17] classify acute kidney injury into three stages of severity (stages 1, 2 and 3).

### Protocol for Paraquat Detoxification

The protocol includes gastric lavage with a large amount of normal saline followed by active charcoal administration, charcoal hemoperfusion, pulse therapies of cyclophosphamide and methylprednisolone followed by dexamethasone therapy, as well as repeated glucocorticoid and cyclophosphamide pulse therapies in case of hypoxemia [2]–[4], [7]–[10].

**Table 7:** Prediction of hospital mortality (n = 187).

Predictive factors	Cutoff point	Youden index	Sensitivity %	Specificity %	Overall correctness %
AKIN_48-h_	1	0.312	61.4	69.8	54.0
SOFA_48-h_	3	0.470	77.2	69.8	73.8
mSOFA_48-h_	4	0.559	73.3	82.6	77.5

Abbreviations. AKIN: acute kidney injury network, SOFA: sequential organ failure assessment, mSOFA: modified sequential organ failure assessment.

### Statistical Analysis

Continuous variables were expressed as mean ± standard deviation. All variables were tested for normal distribution using the Kolmogorov–Smirnov test. The Student’s *t* test was used to compare the means of continuous variables and normally distributed data. Otherwise, the Mann–Whitney *U* test was used for non-normally distributed data. Categorical data were analyzed using a chi-square test. Finally, risk factors were assessed by univariate Cox regression analysis, and variables that were statistically significant (*P*<0.05) were included in a multivariate analysis by applying a multiple Cox regression based on forward elimination of data. Calibration was assessed using the Hosmer–Lemeshow goodness of fit test to compare the number of observed and predicted deaths in risk groups for the entire range of death probabilities. Discrimination was assessed by AUROC. The AUROCs were compared using a non-parametric approach. AUROC analyses were also used to calculate cutoff values, sensitivity, specificity, and overall correctness. Finally, the cutoff points were calculated by acquiring the best Youden index (sensitivity+specificity - 1). The cumulative survival curves as a function of time were generated using the Kaplan–Meier approach and compared by log-rank test. All statistical tests were two-tailed, with *P* values <0.05 being considered as statistically significant. Data were analyzed using SPSS 12.0 software for Windows (SPSS, Inc, Chicago, III).

## Results

### Subject Characteristics

Baseline demographics and clinical characteristics of survivors and non-survivors are shown in Table 3 and 4. The mean age of the patients was 42.1±15.4 years, with 145 (77.5%) being men and 42 (22.5%) women. Overall hospital mortality for the entire population was 54% (101/187). Non-survivors were significantly older, hospitalized more quickly, and had a higher mean ingestion amount of paraquat, blood levels of paraquat, creatinine (Cr), alanine transaminase (AST), and bilirubin upon admission. They also had higher alveolar-arterial differences in oxygen tension (AaDO_2_) 48 h after admission, and had lower partial pressures of carbon dioxide in arterial blood (PaCO_2_)_,_ PaO_2_, HCO_3_^−^, and PaO_2_/FiO_2_ in their arterial blood at the same time point. Survivors and non-survivors differed significantly in their SOFA_48-h_ scores (2±2 versus 4±2, *P*<0.001) (Table 4). Using AKIN_48-h_ classification, the in-hospital mortality was found to be 39.4% (39/99) for stage-0, 64.6% (31/48) for stage-1, 75.0% (12/16) for stage-2, and 79.2% (19/24) for stage-3 patients (*P*<0.001).

### Calibration, Discrimination, and Correlation for SOFA and AKIN Scoring Systems

Calibration of SOFA_48-h_ scores was as carried out as follows: Hosmer–Lemeshow; *X^2^*_2_ = 5.582, *P* = 1.0 (Table 5). AKIN_48-h_ scores also had good calibration, as estimated by the Hosmer–Lemeshow goodness-of-fit test. Table 3 shows the Goodness of Fit for the predicted mortality risk and the predictive accuracy of SOFA_48-h_ and AKIN_48-h_ scores. Table 4 shows the discrimination power of SOFA_48-h_ and AKIN_48-h_ scores. AUROC analysis found that SOFA_48-h_ scores had better discriminatory power for prediction of mortality.

### Clinical Predictors of Mortality

Univariate logistic regression identified several clinical predictors that were significantly associated with mortality (Table 6). Simple linear regression indicated co-linearity between the partial pressure of oxygen in arterial blood 48 h after admission (PaO_2 48-h)_ and SOFA_48-h_ score, and arterial-alveolar differences in oxygen tension at 48 h after admission (AaDO_2 48-h_) and SOFA_48-h_ score. Therefore, PaO_2 48-h_, and AaDO_2__48-h_ were not introduced into the multivariate logistic regression analyses. Multivariate logistic regression analyses identified age, time to hospitalization, blood paraquat level, eGFR_ first day_, and SOFA_48-h_ score were independent predictors of mortality. Notably, the AKIN _48-h_ score was no longer a significant predictor using multivariate analysis. Mortality, sensitivity, specificity, and overall correctness of SOFA_48-h_ and AKIN_48-h_ scores were calculated to determine their predictive value (Table 7). The cumulative survival rates differed significantly (*P*<0.001) between patients with SOFA_48-h_ scores <3 and SOFA_48-h_ scores ≥3 (Fig. 1).

### mSOFA Score

In the multivariate logistic regression model, it was revealed that blood paraquat level was a significant predictor of mortality after intentional paraquat ingestion. Therefore, we recruited this new variable to the original SOFA version, resulting in an mSOFA. Since the mortality prediction of blood paraquat level had a cutoff point of 1.83 ppm (*P*<0.001), in the mSOFA system we assigned patients with blood paraquat levels ≥1.83 ppm one point and those with blood paraquat levels <1.83 ppm zero points. Notably, we found that the mSOFA_48-h_ displayed better discriminatory power (mSOFA_48-h_ vs. SOFA_48-h_, 0.848±0.029 vs. 0.795±0.033, *P*<0.0001) (Table 5) and better sensitivity, specificity, and overall correctness than the original SOFA_48-h_ scores. The cumulative survival rates also differed significantly (*P*<0.001) between patients with mSOFA scores <4 and SOFA_48-h_ scores ≥4. Finally, the survivors and non-survivors differed significantly in their mSOFA scores (2±2 versus 5±2, *P*<0.001) (Table 4). Hence, the mSOFA score was a better predictor of death during hospitalization than the original SOFA score.

## Discussion

In this study, SOFA_48-h_ scores, age, time to hospitalization, blood paraquat levels and eGFR _first day_ were found to be significant predictors of mortality after paraquat poisoning. Many clinical parameters and scoring systems have previously been proposed as mortality predictors for patients with paraquat intoxication [13], [21]–[24]. Models based on SOFA scores employed at admission performed only slightly worse than APACHE II/III scores and were comparable to SAPS II models in predicting patient mortality in general medical and/or surgical ICUs. Models with sequential SOFA scores appear to have comparable performances to other organ failure scores. Cholongitas *et al.* [25] demonstrated that SOFA scores had the best discriminative ability (AUC = 0.79) when compared to APACHE II scores, Model for End-Stage Liver Disease (MELD) scores and King’ s College Hospital (KCH) scores. Craig *et al.* [26] also showed that SOFA scores >7 during the first 96 h post-overdose predicted death/transplantation with a sensitivity of 95.0 (95% CI, 78.5–99.1) and a specificity of 70.5 (95% CI, 66.3–71.6). Apart from these two reports, there have been no other similar studies using SOFA scores to predict mortality for patients with drug overdoses or poisoning, and they have never been used to assess patient outcomes after paraquat intoxication. Therefore, this appears to be the first report demonstrating that SOFA scores >3 are a poor prognosticator for acute paraquat poisoning. Chang *et al.* [21] noted that APACHE II scores >9 had a sensitivity of 64% and a specificity of 88% in predicting the 30-day mortality in acute paraquat-poisoned patients [21]. However, the APACHE II scores do not include indicators of liver damage, which is also a major sequela of paraquat poisoning [10]. The SOFA system is a simple, easily performed, inexpensive, and reproducible scoring method. It is suitable for use in typical hospital wards, where most paraquat patients are admitted. Most importantly, SOFA scores also include parameters of major target organs, such as lung, liver, and kidney. The scoring system applied in this study showed that SOFA scores >3 predict a poorer prognosis compared to those ≤3 (Table 7 and Fig. 1). SOFA scores can therefore assist in predicting the prognosis for paraquat patients and may also assist in the subsequent decision-making processes [2].

Baseline eGFR was also found to be a predictor of mortality in the present study. The AKIN scores were found to be a predictor of mortality by univariate Cox regression analysis, but not after incorporation into a multivariate Cox regression model. At high doses, paraquat can cause acute tubular necrosis, leading to renal failure. As a result, renal excretion of paraquat is markedly reduced, resulting in higher serum concentrations and increased paraquat accumulation in organs, such as the lung and liver. Although renal damage might be reversible if these patients ingested <40 mg/kg paraquat [27], mortality may still occur from delayed pulmonary fibrosis and hypoxemia. Serum paraquat levels were found to be negatively correlated with eGFR in this study using a univariate linear regression model (*P* = 0.012). Lower eGFR in the first 24 h after admission may reflect acute paraquat-induced damage to proximal renal epithelial cells, where active tubular transport occurs [28], [29]. Alternatively, it could reflect a pre-existing reduced renal reserve, which would lead to decreased paraquat elimination and higher paraquat levels in organs and serum. Nevertheless, minor changes in eGFR may not result in upgrading of AKIN scores [17].

Compatible with previous reports, we found that blood paraquat level was the most consistent predictor of mortality after intoxication [15], [30], [31]. However, serum paraquat levels decrease rapidly within the first few hours after ingestion and the time interval between ingestion and serum paraquat measurements is variable between patients. Therefore, the relationship between mortality and serum paraquat levels may be unreliable. Nevertheless, adding serum paraquat level at admission to the original SOFA score can significantly improve the mortality prediction power when assessing patients. Therefore, the mSOFA reflects the key role of the serum paraquat level, which is not included in the original SOFA score. In addition, the increased mortality that was observed for patients who present more quickly at the hospital might be attributable to the fact that patients who ingest more paraquat tend to seek medical attention faster.

In summary, our data demonstrate that either SOFA or mSOFA scores, which are based on the extent of organ function or rate of organ failure, can help to predict mortality after intentional paraquat poisoning. Nevertheless, the retrospective nature of the study, the small patient sample, and the short follow-up time limit the certainty of our conclusions.

## References

[1] Liu SH, Lin JL, Weng CH, Yang HY, Hsu CW, et al (2012) Heart rate-corrected QT interval helps predict mortality after intentional organophosphate poisoning. PLoS One 7: e36576. 22574184 10.1371/journal.pone.0036576 PMC3344908

[2] Tsai TY, Weng CH, Lin JL, Yen TH (2011) Suicide victim of paraquat poisoning make suitable corneal donor. Hum Exp Toxicol 30: 71–73. 20357045 10.1177/0960327110368419

[3] Yen TH, Lin JL, Lin-Tan DT, Hsu CW, Weng CH, et al (2010) Spectrum of corrosive esophageal injury after intentional paraquat ingestion. Am J Emerg Med 28: 728–733. 20637392 10.1016/j.ajem.2009.06.001

[4] Lin JL, Lin-Tan DT, Chen KH, Huang WH (2006) Repeated pulse of methylprednisolone and cyclophosphamide with continuous dexamethasone therapy for patients with severe paraquat poisoning. Crit Care Med 34: 368–373. 16424716 10.1097/01.ccm.0000195013.47004.a8

[5] Talbot AR, Barnes MR (1988) Radiotherapy for the treatment of pulmonary complications of paraquat poisoning. Hum Toxicol 7: 325–332. 3044980 10.1177/096032718800700405

[6] Hampson EC, Pond SM (1988) Failure of haemoperfusion and haemodialysis to prevent death in paraquat poisoning. A retrospective review of 42 patients. Med Toxicol Adverse Drug Exp 3: 64–71. 3285127 10.1007/BF03259932

[7] Lin JL, Leu ML, Liu YC, Chen GH (1999) A prospective clinical trial of pulse therapy with glucocorticoid and cyclophosphamide in moderate to severe paraquat-poisoned patients. Am J Respir Crit Care Med 159: 357–360. 9927343 10.1164/ajrccm.159.2.9803089

[8] Lin JL, Lin-Tan DT, Chen KH, Huang WH, Hsu CW, et al (2011) Improved survival in severe paraquat poisoning with repeated pulse therapy of cyclophosphamide and steroids. Intensive Care Med 37: 1006–1013. 21327593 10.1007/s00134-010-2127-7

[9] Lin JL, Wei MC, Liu YC (1996) Pulse therapy with cyclophosphamide and methylprednisolone in patients with moderate to severe paraquat poisoning: a preliminary report. Thorax 51: 661–663. 8882069 10.1136/thx.51.7.661 PMC472485

[10] Yang CJ, Lin JL, Lin-Tan DT, Weng CH, Hsu CW, et al (2012) Spectrum of toxic hepatitis following intentional paraquat ingestion: analysis of 187 cases. Liver International 32: 1400–1406. 22672665 10.1111/j.1478-3231.2012.02829.x

[11] Lin JL, Liu L, Leu ML (1995) Recovery of respiratory function in survivors with paraquat intoxication. Arch Environ Health 50: 432–439. 8572721 10.1080/00039896.1995.9935979

[12] Sawada Y, Yamamoto I, Hirokane T, Nagai Y, Satoh Y, et al (1988) Severity index of paraquat poisoning. Lancet 1: 1333. 10.1016/s0140-6736(88)92143-5 2897577

[13] Huang NC, Hung YM, Lin SL, Wann SR, Hsu CW, et al (2006) Further evidence of the usefulness of Acute Physiology and Chronic Health Evaluation II scoring system in acute paraquat poisoning. Clin Toxicol (Phila) 44: 99–102. 16615662 10.1080/15563650500514251

[14] Le GallJR, Lemeshow S, Saulnier F (1993) A new Simplified Acute Physiology Score (SAPS II) based on a European/North American multicenter study. JAMA 270: 2957–2963. 8254858 10.1001/jama.270.24.2957

[15] Min YG, Ahn JH, Chan YC, Ng SH, Tse ML, et al (2011) Prediction of prognosis in acute paraquat poisoning using severity scoring system in emergency department. Clin Toxicol (Phila) 49: 840–845. 22077247 10.3109/15563650.2011.619137

[16] Minne L, Abu-Hanna A, de Jonge E (2008) Evaluation of SOFA-based models for predicting mortality in the ICU: A systematic review. Crit Care 12: R161. 19091120 10.1186/cc7160 PMC2646326

[17] Mehta RL, Kellum JA, Shah SV, Molitoris BA, Ronco C, et al (2007) Acute Kidney Injury Network: report of an initiative to improve outcomes in acute kidney injury. Crit Care 11: R31. 17331245 10.1186/cc5713 PMC2206446

[18] Sanchez-Sellero I, Lopez-Rivadulla M, Cruz A, Bermejo A, Fernandez P (1993) A Sequential Spectrophotometric Method for the Determination of Paraquat and Diquat in Plasma. Analytical Letters 26: 1891–1904.

[19] Lin NC, Lin JL, Lin-Tan DT, Yu CC (2003) Combined initial cyclophosphamide with repeated methylprednisolone pulse therapy for severe paraquat poisoning from dermal exposure. J Toxicol Clin Toxicol 41: 877–881. 14677801 10.1081/clt-120025356

[20] Hsu HH, Chang CT, Lin JL (2003) Intravenous paraquat poisoning-induced multiple organ failure and fatality–a report of two cases. J Toxicol Clin Toxicol 41: 87–90. 12645975 10.1081/clt-120018278

[21] Chang MW, Chang SS, Lee CC, Sheu BF, Young YR (2008) Hypokalemia and hypothermia are associated with 30-day mortality in patients with acute paraquat poisoning. Am J Med Sci 335: 451–456. 18552575 10.1097/MAJ.0b013e318157cb6d

[22] Suzuki K, Takasu N, Arita S, Ueda A, Okabe T, et al (1991) Evaluation of severity indexes of patients with paraquat poisoning. Hum Exp Toxicol 10: 21–23. 1673620 10.1177/096032719101000104

[23] Suzuki K, Takasu N, Arita S, Maenosono A, Ishimatsu S, et al (1989) A new method for predicting the outcome and survival period in paraquat poisoning. Hum Toxicol 8: 33–38. 2714808 10.1177/096032718900800106

[24] Scherrmann JM, Houze P, Bismuth C, Bourdon R (1987) Prognostic value of plasma and urine paraquat concentration. Hum Toxicol 6: 91–93. 3817835 10.1177/096032718700600116

[25] Cholongitas E, Theocharidou E, Vasianopoulou P, Betrosian A, Shaw S, et al (2012) Comparison of the sequential organ failure assessment score with the King’s College Hospital criteria and the model for end-stage liver disease score for the prognosis of acetaminophen-induced acute liver failure. Liver Transpl 18: 405–412. 22213443 10.1002/lt.23370

[26] Craig DG, Reid TW, Martin KG, Davidson JS, Hayes PC, et al (2011) The systemic inflammatory response syndrome and sequential organ failure assessment scores are effective triage markers following paracetamol (acetaminophen) overdose. Aliment Pharmacol Ther 34: 219–228. 21554357 10.1111/j.1365-2036.2011.04687.x

[27] Vale JA, Meredith TJ, Buckley BM (1987) Paraquat poisoning: clinical features and immediate general management. Hum Toxicol 6: 41–47. 3546085 10.1177/096032718700600107

[28] Chan BS, Lazzaro VA, Seale JP, Duggin GG (1998) The renal excretory mechanisms and the role of organic cations in modulating the renal handling of paraquat. Pharmacol Ther 79: 193–203. 9776376 10.1016/s0163-7258(98)00015-1

[29] Chan BS, Lazzaro VA, Seale JP, Duggin GG (1996) Characterisation and uptake of paraquat by rat renal proximal tubular cells in primary culture. Hum Exp Toxicol 15: 949–956. 8981098 10.1177/096032719601501202

[30] Jones AL, Elton R, Flanagan R (1999) Multiple logistic regression analysis of plasma paraquat concentrations as a predictor of outcome in 375 cases of paraquat poisoning. QJM 92: 573–578. 10627878 10.1093/qjmed/92.10.573

[31] Proudfoot AT, Stewart MS, Levitt T, Widdop B (1979) Paraquat poisoning: significance of plasma-paraquat concentrations. Lancet 2: 330–332. 89392 10.1016/s0140-6736(79)90345-3

